# Utilizing explainable machine learning for progression-free survival prediction in high-grade serous ovarian cancer: insights from a prospective cohort study

**DOI:** 10.1097/JS9.0000000000002288

**Published:** 2025-01-29

**Authors:** Zhuo Chen, Hui Ouyang, Botao Sun, Jiashan Ding, Yu Zhang, Xinying Li

**Affiliations:** a Department of Gynecology, Xiangya Hospital, Central South University, Changsha, Hunan Province, China; b Gynecological Oncology Research and Engineering Center of Hunan Province, Xiangya Hospital, Central South University, Changsha, Hunan Province, China; c National Clinical Research Center for Geriatric Disorders, Xiangya Hospital, Central South University, Changsha, Hunan Province, China; d National Medical Metabolomics International Collaborative Research Center, Xiangya Hospital, Central South University, Changsha, Hunan Province, China; e Department of General Surgery, Xiangya Hospital, Central South University, Changsha, Hunan Province, China

**Keywords:** machine learning, prediction model, serous ovarian cancer, Shapley additive explanations, Shiny app

## Abstract

**Background:**

High-grade serous ovarian cancer (HGSOC) remains one of the most challenging gynecological malignancies, with over 70% of ovarian cancer patients ultimately experiencing disease progression. The current prognostic tools for progression-free survival (PFS) in HGSOC patients have limitations. This study aims to develop an explainable machine learning (ML) model for predicting PFS in HGSOC patients.

**Methods:**

Nine ML algorithms for PFS prediction were developed using a prospective cohort of 310 HGSOC patients consecutively enrolled from a large Chinese tertiary hospital between January 2017 and December 2020. The optimal model was internally validated using the 1000 bootstrap method. The SHapley Additive exPlanations (SHAP) method was employed to interpret the model in terms of feature importance and feature effects. The final model, constructed with the optimal feature subset, was deployed as an interactive web-based Shiny app.

**Results:**

The random survival forest (RSF) model demonstrated superior predictive performance compared to other ML models, the RFS model constructed with an optimal feature subset in the optimal imputed dataset achieved a superior 1000 bootstrap C-index of 0.755 (95% CI: 0.750–0.780) and a Brier score of 0.183 (95% CI: 0.175–0.190). SHAP analysis identified tumor residual, HE4, FIGO stage, T stage, CA125, age, ascites volume, platelet counts, and BMI as the top nine contributing factors. It also revealed potential nonlinear relationships and important thresholds between HE4, CA125, age, ascites volume, platelet counts, the body mass index, and PFS risk. Additionally, interaction effects were found between tumor residual and age, HE4, and CA125. Finally, an interactive web-based Shiny app for the model was developed and accessible at https://rsfmodels.shinyapps.io/ocRSF/.

**Conclusion:**

An explainable ML model for PFS prediction in HGSOC patients was developed with superior results. The publicly accessible web tool based on the optimized model facilitates its utility in clinical settings, potentially improving individualized patient management and treatment decision-making in HGSOC.

## Introduction

Epithelial ovarian cancer remains one of the most challenging malignancies with limited improvement in mortality over the past decade, emerging as the fourth leading cause of female cancer-related deaths[1]. In 2021, China reported approximately 57 000 new cases of ovarian cancer, with about 39 000 resulting in mortality, reflecting an overall upward trend[2]. Over 70% of ovarian cancer patients ultimately experience disease progression, necessitating further therapeutic interventions, with high-grade serous ovarian cancer (HGSOC) accounting for the majority[3]. Therefore, individualized prediction of progression risk and identification of key prognostic variables are crucial for the management of patients with high-grade serous ovarian cancer.

To date, several studies have developed nomograms to predict progression-free survival (PFS) in ovarian cancer patients^[^4,5^]^. A Cox model-based nomogram developed by Lee *et al* for platinum-sensitive recurrent ovarian cancer included tumor size, platinum-chemotherapy-free interval, CA-125 levels, the number of organ metastatic sites, and the white blood count, achieving a concordance index (C-index) of 0.645[6]. Another study by Tjokrowidjaja *et al* developed a nomogram for BRCA-mutated, platinum-sensitive recurrent ovarian cancer patients undergoing maintenance olaparib therapy, incorporating predictors such as CA-125 levels, platinum-free intervals, measurable disease presence, and the number of prior platinum therapy lines, achieving a C-index of 0.71 in the validation cohort[5]. However, these studies have failed to consider certain important variables, such as age, tumor residual, tumor stage, and HE4 levels, which might be the primary reason for the relatively low predictive performance^[^7–10^]^.

Recently, machine learning (ML) models have shown promise in improving the diagnosis and prognosis of ovarian cancer^[^11,12^]^. These advanced models could detect complex, non-linear relationships between various features and disease outcomes, potentially offering superior predictive capabilities compared to traditional methods. To our knowledge, no ML models have been specifically developed to predict PFS in HGSOC.

Given the limitations of current research and the lack of ML studies on PFS prediction, this study aims to develop and validate an explainable ML model for accurately predicting the individual-level risk of progression in HGSOC. Additionally, we employed the SHapley Additive exPlanations (SHAP) method to identify key predictors and to demonstrate the effects of important features as well as potential interactive effects between features. Moreover, an interactive web-based Shiny app was designed for the model to enhance its applicability in clinical settings.

## Materials and methods

### Patient selection

Our study cohort consisted of 436 patients diagnosed with malignant ovarian tumors between January 2017 and December 2020, who were prospectively and consecutively enrolled, with the final follow-up date in June 2023. The exclusion criteria were as follows: (1) patients not undergoing cytoreductive surgery for ovarian cancer; (2) loss to follow-up postoperatively or unclear treatment processes; and (3) postoperative pathological diagnosis of non-HGSOC. The inclusion and exclusion criteria for cases are illustrated in Supplementary Figure 1, http://links.lww.com/JS9/D843. Standard written informed consent was obtained from all participants or their legal representatives for data collection and publication. STROCCS guidance for the reporting of data was followed[13].

### Candidate variable collection

The candidate variables for the model contained 12 clinicopathological variables, including age, the body mass index before treatment, ascites volume, tumor residual, intraoperative blood loss, FIGO stage, T stage, N stage, M stage, neoadjuvant chemotherapy (NACT), hyperthermic intraperitoneal chemotherapy (HIPEC), and P53 gene mutation, and 19 laboratory diagnostic variables, including lactate dehydrogenase (LDH), human epididymis protein 4 (HE4), carcinoembryonic antigen (CEA), alpha-fetoprotein (AFP), neuron-specific enolase (NSE), β-human chorionic gonadotropin (β-hCG), carbohydrate antigen 125 (CA125), CA199, CA153, CA242, CA724, cytokeratin fragment 19, albumin, alanine aminotransferase (ALT), the white blood cell count (WBC), hemoglobin, the platelet count, serum creatinine (Cr), and blood urea nitrogen (BUN). For variables with missing values, we excluded those with more than 30%[14]. The excluded variables were NSE, β-hCG, CA199, CA153, CA242, CA724, and cytokeratin fragment 19 (Supplementary Figure 2, http://links.lww.com/JS9/D843). Subsequently, five methods were employed for data imputation: mean, histogram, rpart, random forest (RF), and K-nearest neighbor (KNN) (Supplementary Table 1, http://links.lww.com/JS9/D843). The primary outcome was PFS, which was defined as the interval from the initial diagnosis to the recorded recurrence or progression.

### Model and imputed dataset selection

**Figure 1.**
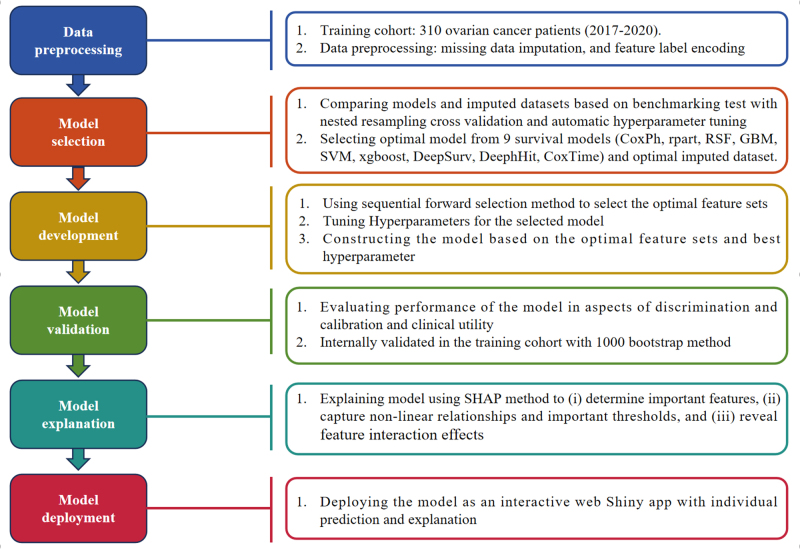
Overview of the study workflow.

**Figure 2.**
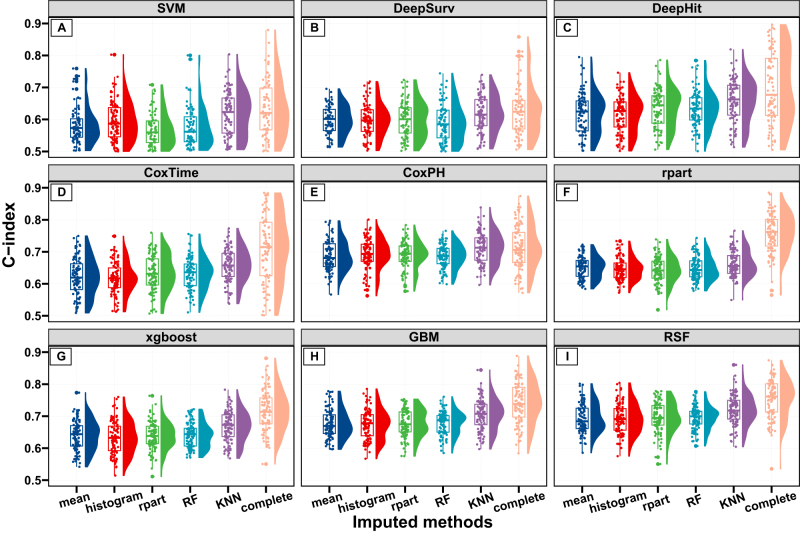
Selection of the optimal model and imputed dataset based on the C-index. Nine machine learning models (A-I) were developed across different datasets (five imputed datasets using mean, histogram, rpart, RF (random forest), KNN (K-nearest Neighbor) methods, and complete case dataset). SVM, support vector machine; RSF, random survival forest; GBM, gradient boosting machine.

The workflow of ML is shown in Fig 1. Nine ML models were developed for predicting PFS in patients with HGSOC: SVM, DeepSurv, DeepHit, CoxTime, CoxPH, rpart, xgboost, GBM, and RSF (Supplementary Table 2, http://links.lww.com/JS9/D843). To obtain an unbiased and objective evaluation of multiple ML models based on different imputed datasets under consistent conditions, a benchmarking test was designed involving nested resampling, automatic hyperparameter tuning, and random search techniques (Supplementary Figure 3, http://links.lww.com/JS9/D843). The selection of the optimal model and dataset was based on a comprehensive evaluation of the C-index and Brier score (Fig. 2, Supplementary Figure 4, http://links.lww.com/JS9/D843, and Supplementary Table 4, http://links.lww.com/JS9/D843). As a result, the RSF model and the KNN imputed dataset were selected for further analysis.

### Feature selection

To enhance the model’s applicability, the sequential forward selection method was employed to select the optimal feature subset based on the selected RSF model and KNN imputed dataset above (Supplementary Figure 5, http://links.lww.com/JS9/D843). Notably, the optimal feature subset was selected by striking a balance between achieving a high C-index and maintaining a manageable number of features (Supplementary Figure 6, http://links.lww.com/JS9/D843 and Supplementary Table 5, http://links.lww.com/JS9/D843).

### Model construction and validation

The final model was developed using the optimal model, imputed dataset, and feature subset above. The performance of the final model was evaluated in terms of discrimination, calibration, and clinical utility. Discrimination ability was measured using the C-index and the time-dependent area under curve (AUC). Calibration capability was assessed through calibration curves and the integrated Brier score. Clinical utility was evaluated using decision curve analysis (DCA). The final model was validated internally using the 1000 bootstrap method. Additionally, a model incorporating all features was developed, and its performance was assessed internally. Notably, the model based on the optimal feature subset was used for web application deployment, while the model incorporating all features was used for model explanation. More details of model construction and validation are provided in Supplementary Methods http://links.lww.com/JS9/D843.

### Model explanation and deployment

To elucidate the inner workings of the RSF model, the SHAP method was applied to the developed model incorporating all features. SHAP summary and dependence plots were used to identify key predictors and investigate their relationships with the outcome. Additionally, SHAP interactive plots were used to identify potential interaction effects between features. To facilitate the accessibility and usability of the model, the final model based on the optimal feature subset was deployed as an interactive web-based Shiny app that enables individualized survival prediction, personalized interpretation, and explanation of the model. More information was described in Supplementary Methods (http://links.lww.com/JS9/D843).

### Sensitivity analysis

Several sensitivity analyses were conducted to assess the robustness of results in the cohort: (1) explaining the RSF model in the dataset with complete information; (2) assessing feature importance using the permutation method; and (3) performing interaction effect analysis with the traditional Cox proportional hazards model.

### Statistical analysis

Summary statistics were presented as total frequencies and percentages for categorical variables and reported as median values with an interquartile range (IQR) or as means with standard deviations (SD) for continuous variables, as appropriate. Differences in data distribution between datasets for both categorical and continuous variables were assessed by the χ2 test and Mann–Whitney *U* test, respectively, with a 2-sided *P*-value < 0.05 considered statistically significant. R version 4.3.1 was used to perform all statistical analysis.

## Results

### Baseline characteristics

The patients’ characteristics are summarized in Table 1. Among the 310 included patients, the mean age was 53.98 years, and the median BMI was 22.67. Patients with FIGO stage I or II disease accounted for 13.5% (42/310) of the total, while the majority (86.5%, 268/310) had advanced stage III or IV disease. Immunohistochemical analysis demonstrated that the vast majority of patients (95.2%, 295/310) had mutated p53 status. At our institution, complete cytoreduction (R0 resection) was achieved in 53.2% (165/310) of patients undergoing cytoreductive surgery, with a median intraoperative blood loss of 400 mL (95% CI 200 to 700 mL). NACT was administered to 26.1% (81/310) of the patients, and 44.2% (137/310) underwent HIPEC. Patients were categorized based on their platinum-free interval following platinum-based chemotherapy, with intervals >6 months and ≤6 months classified as platinum-sensitive and platinum-resistant, respectively. In this cohort, 84.8% (263/310) of the patients were platinum-sensitive at initial chemotherapy, while 15.2% (47/310) developed platinum resistance.

**Table 1:** Patients characteristics in the training cohort

Name	Levels	Total (*N* = 310)	Recurrence		*P*
			No (*N* = 135)	Yes (*N* = 175)	
Age	Mean ± SD	53.98 ± 8.72	53.09 ± 8.87	54.66 ± 8.57	.115
Body mass index	Median (IQR)	22.67 (20.83 to 24.52)	22.55 (20.94 to 24.08)	22.76 (20.83 to 24.86)	.618
T stage*	T1	25 (8.1%)	21 (15.6%)	4 (2.3%)	<.001
	T2	23 (7.4%)	18 (13.3%)	5 (2.9%)	
	T3	262 (84.5%)	96 (71.1%)	166 (94.9%)	
N stage*	N0	214 (69%)	97 (71.9%)	117 (66.9%)	.413
	N1	96 (31%)	38 (28.1%)	58 (33.1%)	
M stage*	M0	250 (80.6%)	115 (85.2%)	135 (77.1%)	.103
	M1	60 (19.4%)	20 (14.8%)	40 (22.9%)	
FIGO staging	Stage I	23 (7.4%)	20 (14.8%)	3 (1.7%)	<.001
	Stage II	19 (6.1%)	15 (11.1%)	4 (2.3%)	
	Stage III	208 (67.1%)	80 (59.3%)	128 (73.1%)	
	Stage IV	60 (19.4%)	20 (14.8%)	40 (22.9%)	
P53 gene mutation	Wild	15 (4.8%)	12 (8.9%)	3 (1.7%)	.008
	Mutation	295 (95.2%)	123 (91.1%)	172 (98.3%)	
Ascites	Median (IQR)	300.00 (50.00 to 2000.00)	200.00 (50.00 to 1000.00)	500.00 (100.00 to 2350.00)	.002
Intraoperative bleeding	Median (IQR)	400.00 (200.00 to 700.00)	400.00 (200.00 to 700.00)	400.00 (200.00 to 750.00)	.493
Tumor reduction	R0	165 (53.2%)	97 (71.9%)	68 (38.9%)	<.001
	R1	103 (33.2%)	33 (24.4%)	70 (40%)	
	R2	42 (13.5%)	5 (3.7%)	37 (21.1%)	
Neoadjuvant chemotherapy	No	229 (73.9%)	115 (85.2%)	114 (65.1%)	<.001
	Yes	81 (26.1%)	20 (14.8%)	61 (34.9%)	
Hyperthermic intraperitoneal chemotherapy	No	173 (55.8%)	78 (57.8%)	95 (54.3%)	.618
	Yes	137 (44.2%)	57 (42.2%)	80 (45.7%)	
Human epididymal protein 4	Median (IQR)	550.25 (241.30 to 946.10)	380.60 (171.56 to 710.10)	708.00 (362.05 to 1180.92)	<.001
Carbohydrate antigen 125	Median (IQR)	786.35 (339.65 to 1635.00)	604.32 (158.40 to 1176.00)	931.50 (486.16 to 1826.00)	<.001
Carcinoembryonic antigen	Median (IQR)	1.06 (0.63 to 1.89)	1.11 (0.65 to 2.05)	1.04 (0.60 to 1.79)	.307
Alpha-fetoprotein	Median (IQR)	2.28 (1.62 to 3.33)	2.18 (1.61 to 3.21)	2.36 (1.68 to 3.45)	.221
Lactic dehydrogenase	Median (IQR)	235.00 (192.00 to 299.00)	224.00 (182.50 to 287.21)	242.00 (194.55 to 311.85)	.074
White blood cell count	Median (IQR)	6.60 (5.40 to 7.90)	6.30 (5.35 to 7.65)	6.80 (5.60 to 7.90)	.257
Hemoglobin	Median (IQR)	119.00 (106.00 to 127.00)	120.00 (107.00 to 127.00)	118.00 (105.64 to 127.00)	.450
Platelet count	Median (IQR)	288.50 (224.00 to 390.00)	263.00 (220.00 to 356.00)	316.00 (245.50 to 406.50)	.005
Albumin	Median (IQR)	38.90 (34.71 to 42.70)	40.40 (35.80 to 43.65)	37.80 (33.70 to 41.95)	.005
Alanine aminotransferase	Median (IQR)	13.80 (9.80 to 19.00)	14.00 (8.70 to 18.60)	13.60 (9.90 to 20.20)	.218
Blood urea nitrogen	Median (IQR)	4.75 (3.73 to 5.90)	4.87 (3.87 to 5.98)	4.71 (3.58 to 5.84)	.354
Blood creatinine	Median (IQR)	67.65 (60.00 to 74.70)	66.56 (58.80 to 73.15)	69.50 (61.00 to 75.50)	.019

FIGO, International Federation of Gynecology and Obstetrics.

^*^ indicated the 8th edition of the American Joint Committee on Cancer (AJCC) T, N, M stage.

### Model selection and construction

A total of nine ML models were developed to predict PFS based on 24 candidate features in five imputed training cohorts. After a benchmarking test (Supplementary Figure 3, http://links.lww.com/JS9/D843), the RSF model in conjunction with the KNN imputed dataset was selected due to its superior predictive performances with the highest mean (SD) C-index of 0.720 (0.047) and the lowest mean (SD) Brier score of 0.146 (0.041) than other models with imputed datasets. Notably, these performance metrics closely approximated those of the RSF model trained on the complete dataset, which achieved a mean (SD) C-index of 0.755 (0.060) and a mean (SD) Brier score of 0.151 (0.039) (Fig. 2, Supplementary Figure 4 http://links.lww.com/JS9/D843, and Supplementary Table 4 http://links.lww.com/JS9/D843). Next, the sequential forward selection method was performed to identify optimal feature subsets that maximized the performance of the RSF model and KNN imputed dataset (Supplementary Figure 5 http://links.lww.com/JS9/D843). As a result, the final model achieving the optimal C-index was constructed by eight features: age, BMI, T stage, FIGO stage, tumor residual, ascites volume, HE4, and CA125 (Supplementary Figure 6, http://links.lww.com/JS9/D843 and Supplementary Table 5, http://links.lww.com/JS9/D843). Additionally, the RSF model with all features was developed for model explanation. Both RSF models, whether utilizing the optimal features or all features, underwent hyperparameter optimization (Supplementary Figures 7 and 8, http://links.lww.com/JS9/D843).

### Model evaluation

**Figure 3.**
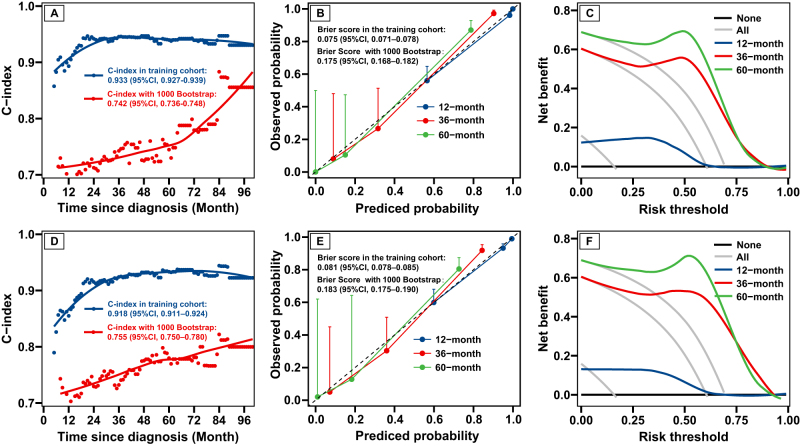
Performance of the RSF model with all features (A, B, C) and the best feature set (D, E, F) in the training cohort. The model performance was comprehensively visualized with the time-dependent area under curve (A, D), calibration plot (B, E), and decision curve analysis plot (C, F). The red lines in plots A and D represent the 1000 bootstrap resampling time-dependent area under curves. RSF, random survival forest.

Fig 3 illustrates the performance of the RSF model using all features (Fig. 3A-C) and the optimal feature subset (Fig. 3D-F) in the KNN imputed dataset. Both models demonstrated excellent discrimination, accuracy, and clinical applicability. The RSF model utilizing all features achieved a C-index of 0.933 (95% CI: 0.927–0.939) in the KNN imputed dataset, while the model with the optimal feature subset attained a C-index of 0.918 (95% CI: 0.911–0.924) (Fig. 3A and 3D, respectively). In internal validation using the 1000 bootstrap method, the RSF model incorporating all features yielded a C-index of 0.742 (95% CI: 0.736–0.748), whereas the final RSF model utilizing the optimal feature subset demonstrated a slightly superior C-index of 0.755 (95% CI: 0.750–0.780) (Fig. 3A and D, respectively). These results indicate that both RSF models exhibit robust discriminative ability, with the model utilizing the optimal feature subset showing marginally improved performance in internal validation. Additionally, the calibration plots were used to assess the predicted accuracy of 1-year, 3-year, and 5-year PFS, revealing a noteworthy correspondence with the ideal curve in both models (Fig. 3B and E). The integrated Brier score for the RSF model with all features was 0.175 (95% CI: 0.168–0.748), while the final RSF model with the optimal feature subset achieved a score of 0.183 (95% CI: 0.175–0.190) in 1000 bootstrap resampling, suggesting the model’s high reliability and accuracy. Moreover, DCA curves affirmed the RSF model’s commendable clinical applicability as a tool for guiding medical intervention (Fig. 3C, F).

### Model explanation

**Figure 4.**
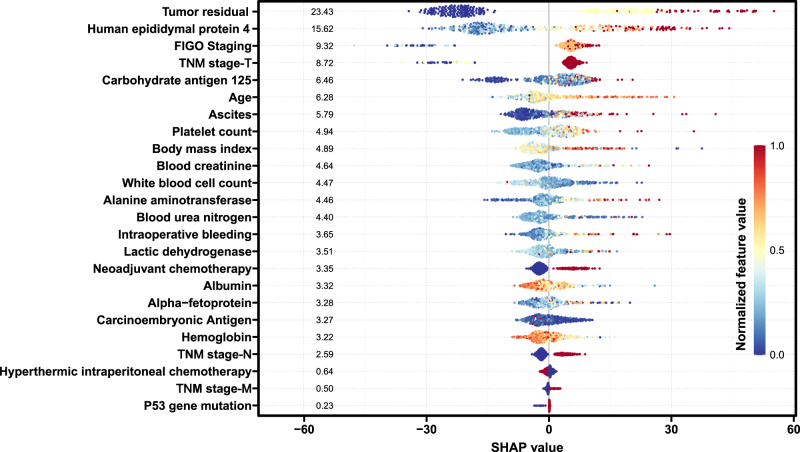
SHAP summary plot of the RSF model with all features in the training cohort. Each dot represented the value of an individual patient data point in the training cohort, with feature’s value ranging from low (in blue) to high (in red). The distance of each dot from the center of the x-axis represents the magnitude of impact (total SHAP value) on the model’s output, with the SHAP value above zero indicating contribution to death (increased death risk) and the SHAP value below zero suggesting contribution to survival (reduced death risk). Features were ranked on the y-axis from the highest to the lowest average contribution (average absolute SHAP value) in terms of feature importance. RSF, random survival forest; FIGO, International Federation of Gynecology and Obstetrics.

In terms of model explanation, the SHAP summary plot was used to identify the key features. As shown in Fig 4, the top nine most important variables contributing to PFS were tumor residual, HE4, FIGO stage, T stage, CA125, age, ascites volume, platelet counts, and BMI. This feature importance ranking showed good consistency with the result in the complete dataset (Supplementary Figure 9, http://links.lww.com/JS9/D843). Moreover, the top nine most important features were further confirmed using the permutation method (Supplementary Figures 15 and 16, http://links.lww.com/JS9/D843), except for albumin, which showed reduced importance in the permutation analysis.

**Figure 5.**
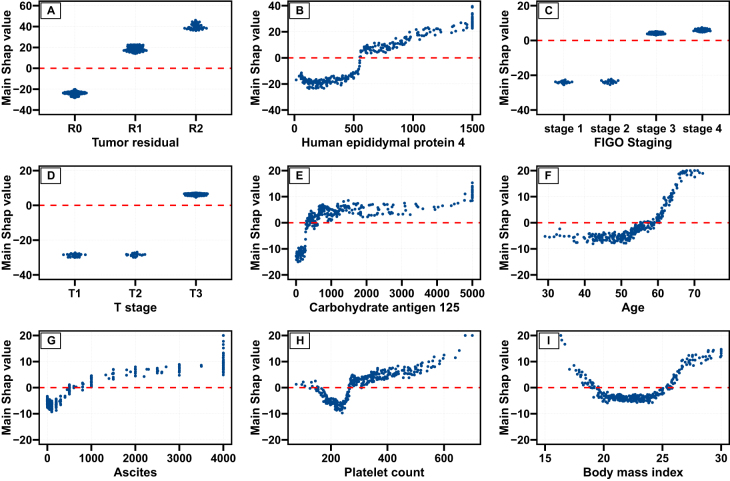
SHAP dependence plot of the top nine important features based on the main SHAP value in the training cohort. Plots showed the main effect of a feature on progression-free survival, including tumor residual (A), human epididymal protein 4 (B), FIGO stage (C), T stage (D), carbohydrate antigen 125 (E), age (F), ascites volume (G), platelet counts (H), and body mass index (I). FIGO, International Federation of Gynecology and Obstetrics.

SHAP dependence plots were used to elucidate the relationships between the top nine important features and the outcome. From the main and total effect plots (Fig. 5 and Supplementary Figure 10, http://links.lww.com/JS9/D843), incomplete cytoreduction, advanced tumor stage, tumor infiltration, and invasion exhibited high SHAP values above zero, suggesting a positive contribution to PFS. Notably, potential nonlinear relationships and important thresholds were observed between HE4, CA125, age, ascites, platelet counts, the body mass index, and PFS risk. Specifically, patients with HE4 levels below 500 pmol/L, age below 55, CA125 below 500 U/mL, and ascites volume below 1000 mL conferred a protective effect. Additionally, there were obvious risk increases with CA125 above 500 U/mL and ascites volume above 1000 mL. Moreover, the U-shaped relationships were observed for the platelet count and BMI, suggesting that both extremes (low/high) for these features are associated with increased PFS risk. Similar results were also observed in the complete dataset (Supplementary Figures 11 and 12, http://links.lww.com/JS9/D843).

**Figure 6.**
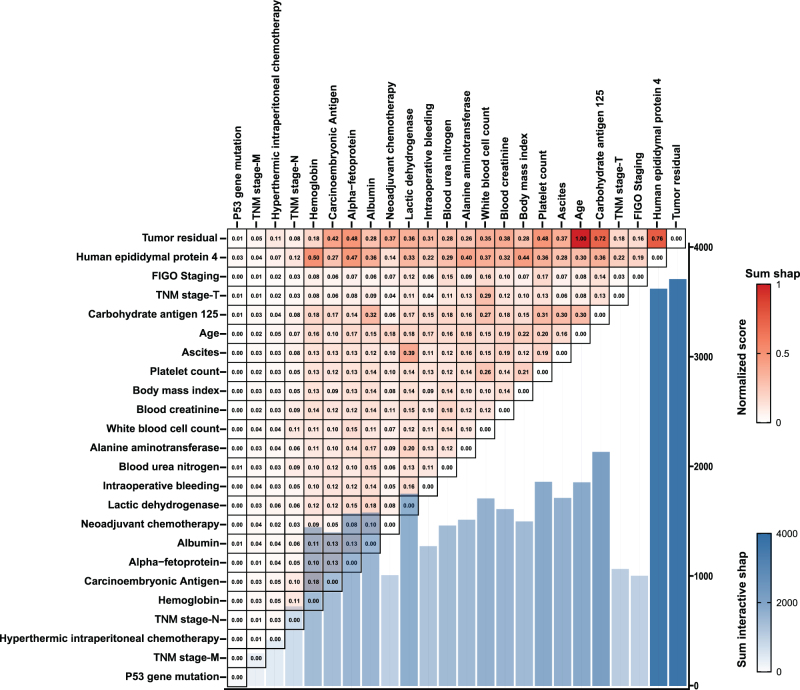
Heatmap of the interaction effect between feature pairs in the training cohort. The value indicated the normalized absolute interaction SHAP value, with a higher score (darker red color) indicating a higher interaction effect between the feature pair. The bar plot suggested the sum absolute interaction SHAP value of a feature with other features, with a darker blue color indicating a higher sum interaction effect. FIGO, International Federation of Gynecology and Obstetrics.

**Figure 7.**
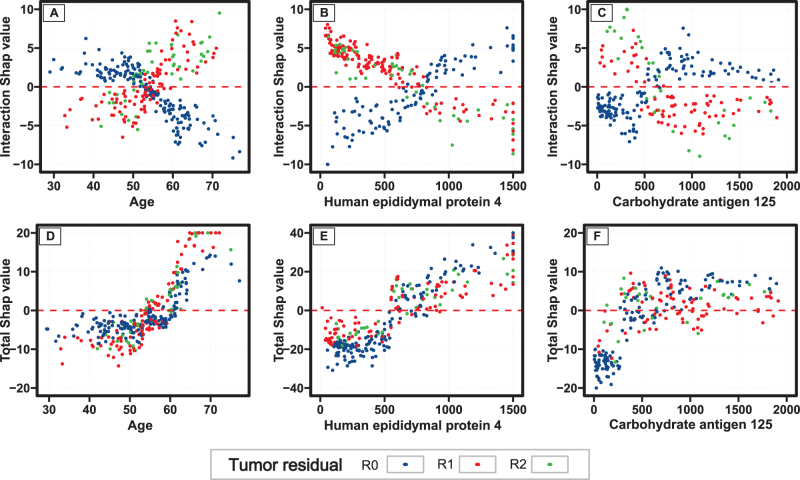
SHAP dependence plot of the three feature pairs based on interaction (A-C) and the total (D-E) SHAP value in the training cohort. Plots visualized the effect of a feature colored by the second feature. Plots A-C indicated the interaction effect of two features on progression-free survival, with a higher interaction SHAP value representing higher addictive positive risk. Plots D-E indicated the interaction effect of the total effect of the two features, and the different color groups of the second feature with vertical dispersion of points (different total SHAP value) at a given x-value indicated the potential interaction effect between feature pairs.

The heat map was used to identify the feature set exhibiting strong interaction effects. As shown in Fig 6, there were strong interactions between tumor residual with age, HE4, and CA125. These interaction patterns were consistently observed in the complete dataset (Supplementary Figure 13, http://links.lww.com/JS9/D843). Fig 7 illustrates the dependence plots for these three feature sets. For instance, Fig 7A reveals a negative interaction (SHAP interaction values < 0, indicating improved prognosis) between age < 55 years and unsatisfactory cytoreduction (R1 or R2). However, for patients aged ≥ 55 years with unsatisfactory cytoreduction, the positive interaction effect suggests that patients aged ≥ 55 years and unsatisfactory cytoreduction would fare worse than expected from the additive prognostic effect of the two variables. Interestingly, the total effect dependence plots (Fig. 7C) further corroborated these interaction patterns, with negative interaction effects (protective effects) observed for age < 55 years and unsatisfactory cytoreduction and positive interaction effects for age ≥ 55 years and unsatisfactory cytoreduction (Fig. 7C). The same interaction pattern was seen in the complete dataset (Supplementary Figure 14, http://links.lww.com/JS9/D843). Moreover, a positive interaction effect (both on multiplicative and additive scales) between tumor residual and age was determined using the traditional Cox model (Supplementary Figure 17, http://links.lww.com/JS9/D843 and Supplementary Table 6, http://links.lww.com/JS9/D843). Similarly, negative interaction effects were observed between residual tumor and HE4 or CA125 in SHAP dependence plots and the traditional Cox model (Fig. 7, Supplementary Figures 14, 18, and 19 http://links.lww.com/JS9/D843, and Supplementary Tables 7 and 8 http://links.lww.com/JS9/D843).

### Model deployment

To enhance the accessibility and practical utility of the final model with the optimal feature subset, an interactive web-based application was developed using Shiny. This application provides individualized survival predictions and explanations, as well as a global interpretation of the model. The web application is accessible at https://rsfmodels.shinyapps.io/ocRSF/.

## Discussion

To our knowledge, this study represents the first comprehensive investigation and comparison of nine ML models for predicting PFS in HGSOC patients. The RSF model emerged as the optimal choice, having been developed and validated with superior predictive performance in terms of discrimination, accuracy, and clinical applicability. Utilizing the SHAP method, we identified the top nine contributing factors associated with increased PFS risk, revealing insightful associations between key predictors and PFS. Additionally, we identified three feature sets that demonstrated strong interaction effects. Furthermore, a publicly accessible web tool was developed for the model enhancing its utility in clinical settings.

Previous studies have predicted PFS in ovarian cancer patients primarily through Cox model-based nomograms, achieving a C-index ranging from 0.645 to 0.710^[^5,6^]^. In contrast, our study has developed the RSF model with eight features and achieved a C-index of 0.918 (95% CI: 0.911–0.924). Importantly, an internal validation using a 1000-guided method yielded a C-index of 0.755 (95% CI: 0.750–0.780). Both of these values surpass the performance of the aforementioned Cox regression nomogram models. A primary explanation for the superior performance of our RSF model may be its incorporation of additional important variables predictive of PFS, such as CA125, HE4, age, and BMI. While previous studies have suggested that these variables may influence PFS, our findings further confirm their importance and predictive significance in the context of ovarian cancer. The integration of CA125 and HE4 as biomarkers is particularly noteworthy. CA125 is a well-established tumor marker in ovarian cancer, commonly used to monitor treatment response and disease progression. HE4 has emerged as a promising biomarker that may provide additional insights, particularly in distinguishing between malignant and benign pelvic masses[15]. By including both biomarkers, our model capitalizes on their complementary roles in assessing disease status. Incorporating patient-specific factors such as age and BMI further enhances the model’s relevance. Age is a well-documented prognostic factor in ovarian cancer, with older patients often experiencing poorer outcomes due to various clinical and biological factors[16]. Similarly, BMI has been associated with treatment responses and overall prognosis in cancer patients[17]. By considering these variables, our model provides a more nuanced understanding of PFS in ovarian cancer patients.

Another key factor contributing to the enhanced predictive capability of our model is the inherent strength of machine learning approaches in identifying complex, non-linear relationships that traditional Cox regression methods may overlook[18]. This assertion is supported by our model explanation results, which revealed potential non-linear relationships among HE4, CA125, age, BMI, and PFS. Furthermore, we observed a stronger interaction effect between residual tumor presence and the variables of age, HE4, and CA125, indicating that these factors may work synergistically to influence patient outcomes. Overall, our results highlight the potential of the RSF model as a powerful tool for predicting PFS in HGSOC patients, surpassing the performance of traditional Cox-based nomograms. The model’s ability to integrate key biomarkers and patient-specific factors, along with its capacity to detect complex interactions, underscores its clinical applicability.

In our analysis of key predictors affecting PFS in ovarian cancer patients, we identified nine critical variables by order: residual tumor, HE4, FIGO stage, T stage, CA125, age, ascites volume, platelet counts, and BMI. Concerning tumor residual, prior research strongly indicated that satisfactory cytoreduction is crucial for improving PFS and overall survival (OS)^[^19,20^]^. Our findings align with this, revealing a negative correlation between postoperative residual tumor burden and PFS. This factor ranks highest among all clinical features, reinforcing the importance of aggressive surgical intervention. Despite ongoing debates regarding the efficacy of suboptimal cytoreduction, our study confirms the value of clinical physicians’ efforts to achieve satisfactory cytoreduction within the permissible range of surgical conditions^[^21,22^]^. This highlights the need for a nuanced approach to surgical strategies in HGSOC treatment, emphasizing that any degree of tumor reduction may confer benefits to patient outcomes.

A particularly groundbreaking finding from our study is the significant impact of preoperative plasma HE4 levels on prognosis, which appears to surpass even tumor staging. A recent study has indicated an association between abnormal preoperative HE4 levels and the risk of residual disease post-surgery[23]. Another study revealed higher HE4 levels in wild-type ovarian cancer patients compared to those with BRCA1/2 mutations, correlating with micronodular carcinomatosis and a poor prognosis[24]. Moreover, elevated HE4 levels may indicate primary resistance to platinum-based chemotherapy in BRCA1/2 mutation carriers[25]. Similarly, our study demonstrates that high plasma HE4 levels are a significant factor in influencing PFS. Notably, the nonlinear effect of HE4 levels on prognosis suggests the existence of a potentially valuable predictive threshold. However, the molecular mechanisms by which HE4 influences tumor progression remain incompletely understood, highlighting the need for further prospective analyses focused on HE4 levels[7].

In our analysis of interactions, an important finding was the observation of a positive interaction effect between tumor residual and age. Specifically, patients ≥55 years old who underwent R1/R2 cytoreductive surgery exhibited a worse prognosis than would be expected based on the additive effects of these two risk factors. Both age ≥ 55 and R1/R2 cytoreductive surgery were identified as independent risk factors for PFS. This positive interaction effect between them highlights the compounded risk that older patients face when tumor residual burden is present following surgery. It suggests that the impact of age on prognosis may be exacerbated by the presence of tumor residual, indicating that older patients with suboptimal surgical outcomes may require more intensive monitoring and potentially more aggressive treatment strategies. Therefore, understanding this interaction is crucial for clinical decision-making, as it underscores the need for tailored approaches to treatment in older patients with ovarian cancer.

Finally, to further enhance clinical applicability, we developed a web-based interactive application using Shiny. The development of this interactive web application represents a significant step towards translating our research findings into practical clinical applications. By enabling healthcare providers to generate personalized survival estimates and assess the impact of key variables, this tool empowers clinicians to make more informed treatment decisions tailored to each patient’s specific risk profile. In our opinion, this level of personalization is crucial in ovarian cancer management, where individual patient characteristics can significantly influence treatment outcomes.

Our study has several limitations that should be acknowledged. First, due to some patients being referred from tertiary centers, pre-treatment imaging data were not complete, resulting in the exclusion of imaging evidence prior to treatment. This lack of comprehensive imaging data may limit the robustness of our findings. Second, the data used in this study were sourced from a single center, which lacks external validation. This limitation may introduce potential bias and restrict the generalizability of our results to broader populations. Finally, recent research has highlighted the potential significance of molecular biomarkers such as BRCA1/2, homologous recombination status (HR status), microsatellite instability (MSI), mismatch repair (MMR), and tumor mutational burden (TMB) in ovarian cancer[26]. However, the omission of these important biomarkers may limit the predictive accuracy of our model. To address these limitations, future research should focus on larger-scale, multicenter cohorts to validate our findings and incorporate molecular testing results to improve the model’s predictive performance.

## Conclusion

This study presents the first comprehensive comparison of machine learning models for predicting PFS in HGSOC, with the RSF model demonstrating superior performance. Our explainable model integrates key biomarkers and clinical factors, highlighting the significance of tumor residual, HE4, FIGO stage, T stage, CA125, age, ascites volume, platelet counts, and BMI in prognosis. The identification of an interaction effect between age and tumor residual underscores the need for tailored treatment strategies in older patients. Additionally, we developed a web-based Shiny application to facilitate personalized survival predictions, enhancing the clinical utility of our findings and supporting informed decision-making in patient management.

## Data Availability

The datasets used and/or analyzed for the present study are available from the corresponding author on reasonable request.

## Research registration unique identifying number (UIN)

Research Registry (researchregistry10766).
